# Trends and inequality of human resource in centers for disease control and prevention in China from 2019 to 2023

**DOI:** 10.3389/fpubh.2025.1553314

**Published:** 2025-04-16

**Authors:** Na Zhang, Qiong Wu, Shaoqiong Li, Chongyi Wang, Ayan Mao, Qing Guo, Wuqi Qiu

**Affiliations:** ^1^Institute of Medical Information, Chinese Academy of Medical Sciences and Peking Union Medical College, Beijing, China; ^2^Information Center, Chinese Center for Disease Control and Prevention, Beijing, China

**Keywords:** public health workforce, inequality, trend, Gini coefficient, Theil index

## Abstract

**Background:**

The public health workforce plays a crucial role in the development of health systems, particularly in enhancing the capacity of public health infrastructure. Understanding the current status of human resources in the Centers for Disease Control and Prevention (CDC) is essential for establishing future development goals. This study aims to assess the quantity and equity of the human resources in China’s CDCs since the outbreak of COVID-19, with the goal of promoting a more equitable distribution of the CDC workforce and enhancing the capacity to respond effectively to major public health emergencies.

**Methods:**

Using nationwide administrative data from China CDC (2019–2023), we conducted a two-stage analysis: First, we conducted a descriptive analysis of the current status and trends of the CDC workforce. Second, we performed an equity assessment through multilevel decomposition (1) Calculated Gini coefficients across three dimensions (geography, Gross Domestic Product per capita, population density); (2) Applied Theil T/L index to partition inequalities into within-region and between-region components.

**Results:**

Between 2019 and 2023, the CDC workforce in China increased to 230,594 employees, with workforce density rising from 1.3 to 1.64 per 10,000 residents, 76.26% being health professionals, a majority of whom were female, and the 25–34 age group comprising the largest segment (29.06%). Additionally, while the proportion with less than 5 years of service grew, staff with over 30 years of service formed the largest tenure group (30.69%). The Gini coefficient revealed extreme spatial inequality, indicating that geographic distribution was significantly exceeding those based on economic and population distributions, with values of 0.5815, 0.3866, and 0.1843, respectively, in 2023. Decomposition of inequality indicated that both general staff and health professionals were primarily affected by within-region disparities, with an increasing trend of within-region inequity from 2019 to 2023. In 2023, Theil T analysis showed that within-region inequality accounted for 76.67% of staff disparities, while for health professionals, this figure was 64.21%.

**Conclusion:**

The human resource landscape in China’s CDCs faces challenges related to both underfunding and an aging workforce. Inequities in workforce distribution persisted from 2019 to 2023, with significant disparities within regions. Strengthening the CDC workforce, particularly in underdeveloped and sparsely populated areas, is essential for addressing these challenges.

## 1 Introduction

Building public a health workforce, capable of delivering the full range of essential health services and basic public health functions, is the cornerstone of a resilient health system to achieve and sustain universal health coverage, health security, and health-related sustainable development goals (1, 2). Whether in the context of preparing for, preventing, and responding to emerging infectious disease outbreaks, or addressing other diverse challenges such as climate change, the escalating burden of non-communicable diseases (3), and antimicrobial drug resistance, it is imperative to strengthen the public health workforce (4).

Following the SARS epidemic, China prioritized strenthening its public health system. A network of Centers for Disease Control and Prevention (CDC) was established at provincial, prefectural and county levels, which constitutes the core of China’s public health system. Workforce management within the CDC has been central to these efforts (5, 6). In 2009, China initiated a new round of healthcare reform, making the public health service system one of the four core reform areas, with an emphasis on the allocation of public health personnel (7).

Concrete workforce standards emerged in subsequent years. In 2014, a standard was set to determine the number of CDC staff based on a ratio of 1.75 per 10,000 residents, with higher ratio of 3 per 10,000 in sparsely populated areas. This standard has been in use ever since (8). In 2016, a goal was set to have more than 0.83 professional public health institution personnel per 1,000 residents (9). Despite these preparatory measures, the COVID-19 pandemic exposed chronic workforce shortages and operational overload (10). China’s rapid issuance of reinforcing directives during the crisis enabled relatively effective containment (11–13), largely through the operational pivot of CDC teams—particularly epidemiological investigators—who became the frontline defense (14, 15).

However, the pandemic also exposed several weaknesses in the disease prevention and control system (16). In response, a talent development plan was launched in 2022, setting a target to increase the CDC workforce to 250,000 by 2025, offering a more detailed staffing blueprint for the CDCs (17). In the same year not long after, China issued the “Guiding Opinions on Promoting the High-Quality Development of Disease Prevention and Control” at the end of 2022, marking the start of reforms aimed at enhancing the quality of the talent pool in the disease prevention and control system (18). But quantitative analyses of workforce fluctuations during and post-pandemic remain scarce. Systematically tracking workforce changes across 2019–2023 is essential to evaluate policy efficacy, identify disparities across regions, and inform future workforce optimization strategies.

This challenge transcends national boundaries. Data shows that the United States has also faced public health workforce shortages in recent years, compounded by high personnel turnover. Public health workers in the U.S report high levels of job burnout and a strong desire to leave their positions (19, 20). In response, the U.S. has launched significant funding initiatives to strengthen its public health workforce (21, 22).

Within China, understanding CDC workforce trends requires contextualizing national targets against global benchmarks while addressing persistent attrition rates. Existing studies are lack of comprehensive post-COVID analyses. This study bridges that gap by leveraging data from the “China National Basic Information System for Disease Prevention and Control” (CNBIS), analyzing provincial-level workforce distribution and quantitative shifts across the pandemic era (2019–2023). Such evidence-based inquiry informs both domestic policy refinement and global knowledge exchange in public health workforce development.

## 2 Theoretical analysis—Working Lifespan Approach

This study adopts the World Health Organization’s Working Life Approach, which provides a comprehensive framework for understanding the dynamic nature of health workforce (23). The approach conceptualizes the career trajectory of health professionals in three distinct phases: entry (recruitment and initial training), active service (retention, skill development, and service delivery), and exit (attrition due to retirement, career changes, or other factors) (24). By framing the analysis within this lifecycle model, we capture both quantitative trends in the CDC workforce over time and qualitative aspects of workforce composition and equity.

The relevance of this framework to our study is twofold. First, it supports an assessment of workforce trends by highlighting critical indicators such as total staff numbers, age distribution, and years of service. These indicators reflect the different stages of the working lifespan and offer insights into the current status and future trajectory of China’s CDC human resources from 2019 to 2023 (23). Second, by integrating equity measures, such as the Gini coefficient and Theil index, our analysis addresses disparities in the geographic, economic, and population-based distribution of CDC personnel. This equity assessment is essential for identifying imbalances that may hinder the CDCs’ capacity to respond effectively to public health emergencies (25).

Moreover, the application of the Working Lifespan Approach aligns with existing research that emphasizes balanced recruitment, retention, and attrition as critical determinants of a resilient public health workforce. Prior studies have demonstrated that uneven workforce distribution not only undermines operational efficiency but also compromises the effectiveness of health service delivery, especially during crisis like the COVID-19 pandemic (25–27). By adopting this theoretical perspective, our study contributes to broader debates on sustainable health workforce development and equitable resource allocation.

## 3 Data and methods

### 3.1 Data source

We obtained year-end data on the number of public health workers (CDC human resources) in China from 2019 to 2023 from the CNBIS, a database established by the national CDC. As an annual, organization-based administrative survey system, the CNBIS collects demographic data on CDC staff, human resource capacity, and other relevant information. The system serves as a critical tool for public health planning, performance assessment, and resource allocation. It covers all provincial, prefecture and county level CDCs in China.

### 3.2 Measures

We analyzed two categories of CDC human resources:

(1) Staff, which were all individuals employed at CDCs, including both permanent and temporary staff;
(2) Health professionals, a subset of staff with specific health expertise and operational skills, including physicians, registered nurses, pharmacists, imaging technicians and laboratory examination technicians.

Health regions refer to the eastern, central and western regions of China, divided by the National Health Commission of China (28).The eastern region includes Beijing, Tianjin, Hebei, Liaoning, Shanghai, Jiangsu, Zhejiang, Fujian, Shandong, Guangdong and Hainan. The central region comprises Shanxi, Jilin, Heilong Jiang, Anhui, Jiangxi, Henan, Hubei, and Hunan. And the western region covers Inner Mongolia, Chongqing, Guangxi, Sichuan, Guizhou, Yunnan, Tibet, Shaanxi, Gansu, Qinghai, Ningxia, and Xinjiang Uygur Autonomous Region.

### 3.3 Statistical analysis

We calculated the density of CDC staff (per 10,000 population) using workforce data from CNBIS and population data from the *China Statistical Yearbook (2020–2024)* (29). The density metric was calculated using the standardized formula: *Density = Total staff/10,000 population.*

To analyze temporal trends in workforce distribution, we applied linear regression modeling to CDC density data spanning 2019–2023. Complementing this temporal analysis, we implemented a tripartite inequality assessment framework comprising: Gini coefficient, Theil T, and Theil L. This methodological approach aligns with the World Health Organization’s technical guidelines outlined in “*Measuring health workforce inequalities: methods and application to China and India”* (2010), which advocates the concurrent use of these three metrics to establish standardized evaluations of health workforce distribution (30). The Gini coefficient is a classical and extensively used measure in equity analysis, and provides a single summary statistic of disparity (31–33). The Theil index, validated by the World Health Organization (34), is essential for identifying the primary drivers of inequity in the CDC workforce, thereby providing nuanced insights that can inform targeted policy interventions. The Theil index is decomposable, and the Theil T and Theil L measures were both used in this study to assess overall inter-provincial inequality, broken down into within-region and between-region components. Descriptive and equity analyses were conducted using R 4.3.0 and Microsoft Excel 2019.

#### 3.3.1 Gini coefficient

The Gini coefficient was originally developed by Corrado Gini in 1912 (35), ranging from 0 (perfect equality) to 1 (maximum inequality). While it is not inherently decomposable, its simplicity and widespread use make it a valuable complementary tool in inequality analysis. The coefficient is calculated based on the Lorenz curve, where the area between the curve and the line of equality is divided by the total area under the line of equality:


$$G=\frac{1}{2N^{2}}\sum_{i=1}^{N}\sum_{j=1}^{N}|x_{i}-x_{j}|$$

Here, $x_{i}$ and $x_{j}$ represent the health worker densities in units i and j, and N is the total number of units.

#### 3.3.2 Theil L index

The Theil L index, introduced by Henri Theil in 1967 (36), is a decomposable entropy measure that quantifies the divergence between observed health worker shares and population shares across geographical units. It is particularly suited for hierarchical decomposition into within-group and between-group inequality components. The index is defined as:


$$L=\sum_{i=1}^{N}\left(\frac{p_{i}}{P}\right)\ln\left(\frac{X}{x_{i}}\right)$$

In this equation, $p_{i}$ is the population in unit i, P is the total population, $x_{i}$ is the health worker density in unit i, X is the national health worker density (H/P), ln denotes the natural logarithm.

This formula can also be expressed as the difference between the logarithm of the arithmetic mean (*X*) and the logarithm of the geometric mean (*χ*) of health worker densities:


$$L=\ln\left(X\right)-\ln\left(\chi \right)=\ln\left(\frac{X}{\chi }\right)$$

The Theil L index is strictly additively decomposable, allowing the partitioning of overall inequality into contributions from within-group and between-group differences. However, it is undefined when any unit has zero health workers, as this leads to an infinite geometric mean (*χ* = 0).

#### 3.3.3 Theil T index

The Theil T index, also proposed by Theil (1967) (36), is another entropy-based measure that reverses the roles of health worker shares and population shares in the Theil L formula. It is defined as:


T=∑i=1NhiHlnhiHpiP

In this equation, $h_{i}$ is the number of health workers in unit *i,*H is the total number of health workers, $p_{i}$ and P are as defined earlier.

The Theil T index is well-defined even when some units have zero health workers, as terms with zero health worker shares ( $h_{i}$ = 0) contribute zero to the sum ( xlnx →0 as x →0). However, its decomposition properties are weaker than those of the Theil L index. Specifically, the within-group component of the Theil T index does not correspond to the strict additive decomposition of inequality, making it less suitable for detailed hierarchical analysis.

In this study, the Theil L index was primarily used due to its strong decomposition properties, enabling the isolation of within-province and between-province inequality contributions. The Theil T index was employed as a robust alternative when zero health worker densities are present. The Gini coefficient was included for comparative purposes and to align with conventional inequality metrics in policy and research contexts. These measures collectively provided a comprehensive framework for analyzing and addressing maldistribution in health workforce.

## 4 Results

### 4.1 Basic information on CDC workforce in China

As of 2023, China’s CDC workforce consisted of 230,594 staff members, 76.26% of whom were health professionals. Women outnumbered men, and the largest age group was individuals aged 25–34 years. The largest proportion of CDC workforce had over 30 years of service. From 2019 to 2023, the number of CDC staff per 10,000 residents increase from 1.30 to 1.64 (P < 0.01). The proportion of health professionals also increased, from 71.92% in 2019 to 76.26% in 2023. Detailed data are shown in Table 1.

**Table 1:** Basic information of CDCs human resource in China, 2019–2023.

	2019	2020	2021	2022	2023	*p* for trend
Total	183,820	194,535	205,384	224,205	230,594	<0.001
density(total person/10,000 population)	1.30	1.38	1.45	1.59	1.64	0.001
Percent of health professionals (%)	71.92	72.41	72.83	73.25	76.26	0.048
Gender (%)						
Male	44.43	43.84	43.03	41.86	41.29	<0.001
Female	55.57	56.16	56.97	58.14	58.71	<0.001
Age (%)						
<25 years old	2.75	3.01	3.71	4.66	4.13	0.045
25–34 years old	24.03	24.23	25.11	27.28	29.06	0.011
35–44 years old	30.82	29.66	28.5	27.52	27.00	0.001
45–54 years old	32.38	31.85	30.85	29.48	28.76	0.001
≥55 years old	10.02	11.25	11.83	11.05	11.06	0.442
Years of service (%)						
<5 years	12.02	12.98	15.24	19.28	21.12	0.003
5–9 year	12.26	12.17	12.19	12.23	12.07	0.191
10–19 year	20.57	21.02	21.22	21.49	22.33	0.008
20–29 year	29.69	28.28	26.02	23.63	22.49	<0.001
≥30 year	21.09	23.12	24.2	24.02	23.76	0.122

In terms of gender structure, the proportion of male staff declined from 44.43% in 2019 to 41.29% in 2023, while female representation increased from 55.57% to 58.71%. Regarding age structure, the proportion of workers aged 35–44 years and 45–54 years declined, while the percentage of workers aged younger than 25 years and 25–34 years increased. In 2023, the highest percentage of staff were aged 25–34 years (29.06%), followed by those aged 45–54 years (28.76%). The smallest proportion was made up of workers under 25 years (4.13%).

In terms of years of service, the percentage of workers with less than 5 years of service increased from 2019 to 2023, while the proportion with 10–19 years and 20–29 years of service decreased. As of 2023, the largest proportion of CDC staff (30.69%) had 30 or more years of service, followed by those with 20–29 years and 10–19 years. The proportions of staff with less than 5 years, 10–19 years, and 20–29 years of service were relatively close, at 21.12%, 22.33%, and 22.49%, respectively.

### 4.2 Distribution

#### 4.2.1 Gini coefficient

The Gini coefficients for population, economic and geographical distribution showed minimal variation from 2019 to 2023. Geographically, the Gini coefficient was the highest, followed by that of economic distribution, while the population distribution had the lowest value.

**Figure 1:**
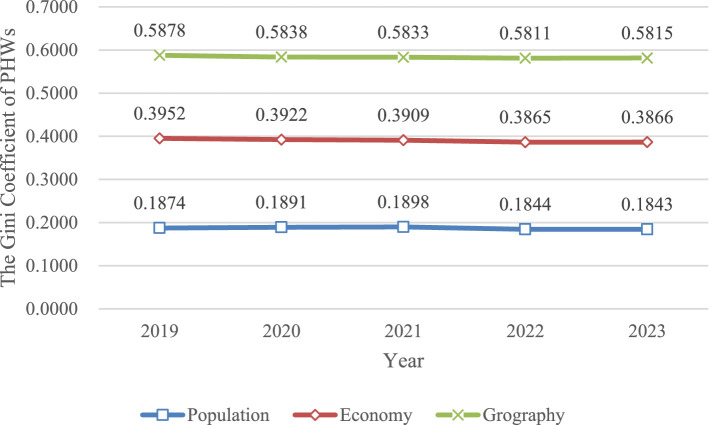
Trend of Gini coefficient in population, economic, geographical dimensions of Chinese public health workforce in 2019–2023.

From 2019 to 2023, the Gini coefficient for the population distribution of staff in China ranged from 0.1843 to 0.1898, with all three regions showing coefficients below 0.2. The Gini coefficient for economic distribution fluctuated between 0.3865 and 0.3952, slightly below the 0.4 threshold. The Gini coefficient for geographic distribution varied between 0.5811 and 0.5878, remaining above the 0.4 threshold. Notably, the western region consistently exceeded this threshold, reaching 0.5457 in 2023, while the eastern and central regions were well below 0.4 (0.2799 and 0.3000 respectively, in 2023). Figure 1 and Table 2 provide further details.

**Table 2:** Gini coefficient for CDC human resource by population, economy, and geography across different regions in China, 2019–2023.

Year	**Staff**									**Health professionals**								
	**Population**			**Economy**			**Geography**			**Population**			**Economy**			**Geography**		
	**Eastern region**	**Central region**	**Western region**	**Eastern region**	**Central region**	**Western region**	**Eastern region**	**Central region**	**Western region**	**Eastern region**	**Central region**	**Western region**	**Eastern region**	**Central region**	**Western region**	**Eastern region**	**Central region**	**Western region**
2019	0.1521	0.1300	0.1084	0.3828	0.2395	0.3812	0.2646	0.3351	0.5731	0.0860	0.1161	0.1209	0.4026	0.1832	0.3838	0.2462	0.2977	0.5707
2020	0.1480	0.1342	0.1214	0.3805	0.2425	0.3785	0.2702	0.3227	0.5621	0.1013	0.1162	0.1343	0.4029	0.1898	0.3819	0.2454	0.2935	0.5586
2021	0.1564	0.1387	0.1323	0.3882	0.2572	0.3741	0.2884	0.3165	0.5518	0.1090	0.1225	0.1437	0.4158	0.2067	0.3777	0.2566	0.2862	0.5486
2022	0.1579	0.1326	0.1330	0.3914	0.2519	0.3673	0.2891	0.3029	0.5472	0.1075	0.1167	0.1420	0.4193	0.2048	0.3731	0.2492	0.2790	0.5438
2023	0.1672	0.1384	0.1344	0.3893	0.2564	0.3599	0.2799	0.3000	0.5457	0.1174	0.1157	0.1418	0.4157	0.2146	0.3647	0.2309	0.2812	0.5432

**Figure 2:**
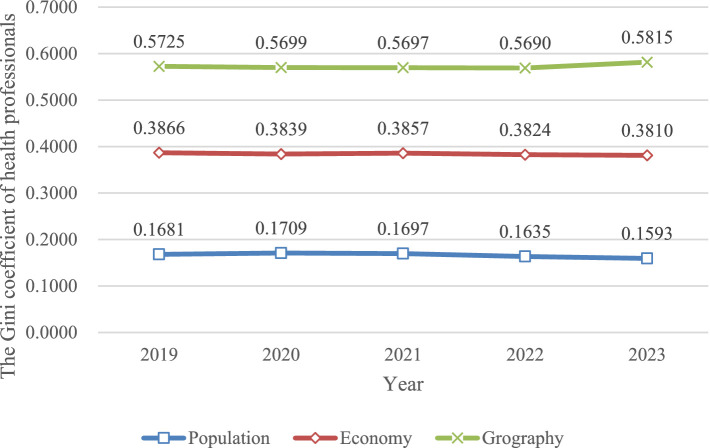
Trend of Gini coefficient in population, economic, geographical dimensions of health professionals in 2019–2023.

The Gini coefficient for health professionals mirrored that of the staff, with the population distribution Gini coefficient remaining under 0.2, the economic distribution Gini coefficient under 0.4, and the geographic distribution Gini coefficient exceeding 0.4. The western region again had the highest Gini coefficient for geographic distribution, surpassing 0.5 (0.5432 in 2023), while the eastern and central regions were below 0.4 (0.2309 and 0.2812, respectively, in 2023). Figure 2 illustrates these details.

#### 4.2.2 Theil T and Theil L index

From 2019 to 2023, the overall Theil T and L index for both CDC staff and health professionals changed little. Notably, the Theil T for staff was higher than that for health professionals, with both index showing similar trends.

For CDC staff, the Theil T fluctuated between 0.0559 and 0.0587 from 2019 to 2023, while the Theil L fluctuated between 0.0543 and 0.0564. There were regions difference: the eastern region had the highest Theil T and L, with a Theil T of 0.0581 in 2023, while the central and western regions had lower values (0.0323 and 0.0349, respectively). The Theil L for these regions values were 0.0507, 0.0312, and 0.0327, respectively.

For health professionals, the Theil T fluctuated between 0.0447 and 0.0476 from 2019 to 2023, and the Theil L ranged from 0.0426 to 0.0455. Regional disparities were also evident: the western region had the highest Theil T and L, with 0.0373 for Theil T and 0.0353 for Theil L in 2023, while the eastern and central regions had lower values (0.0232 and 0.0254 for Theil T, 0.0235 and 0.0353 for Theil L). Table 3 presents these findings.

**Table 3:** Theil T and Theil L index for CDC human resource across different regions in China from 2019–2023.

**Year**	**Staff**								**Health professionals**							
	**Theil T**				**Theil L**				**Theil T**				**Theil L**			
	**Overall inter-province**	**Eastern region**	**Central region**	**Western region**	**Overall inter-province**	**Eastern region**	**Central region**	**Western region**	**Overall inter-province**	**Eastern region**	**Central region**	**Western region**	**Overall inter-province**	**Eastern region**	**Central region**	**Western region**
2019	0.0559	0.0542	0.0290	0.0226	0.0556	0.0449	0.0309	0.0236	0.0457	0.0135	0.0217	0.0267	0.0438	0.0129	0.0215	0.0280
2020	0.0571	0.0538	0.0296	0.0272	0.0560	0.0440	0.0305	0.0287	0.0472	0.0174	0.0221	0.0320	0.0454	0.0172	0.0296	0.0339
2021	0.0587	0.0592	0.0317	0.0313	0.0564	0.0480	0.0323	0.0317	0.0476	0.0193	0.0251	0.0354	0.0455	0.0197	0.0240	0.0360
2022	0.0575	0.0580	0.0294	0.0345	0.0543	0.0479	0.0288	0.0329	0.0461	0.0190	0.0250	0.0372	0.0434	0.0197	0.0233	0.0361
2023	0.0575	0.0581	0.0323	0.0349	0.0552	0.0507	0.0312	0.0327	0.0447	0.0232	0.0254	0.0373	0.0426	0.0242	0.0235	0.0353

The decomposition of the Theil T and L index revealed that both CDC staff and health professionals were dominated by within-region inequality. Furthermore, from 2019 to 2023, within-region inequality increased, while between-region inequality decreased. In addition, the share of within-region inequality was higher for CDCs staff than for health professionals. In 2023, within-region inequality accounted for 76.67% of the Theil T for staff and 64.21% for health professionals. For the Theil L index, the corresponding values were 72.51% and 63.36%, respectively. These findings are summerized in Tables 4, 5.

**Table 4:** Regional attribution of Theil T and Theil L for CDC human resource in China, 2019–2023.

Year	**Theil T**					Theil L				
	**Overall inter-province inequality**	**Within- regional inequality**	**percentage of within- regional inequality**	**Between- regional inequality**	**percentage of between- regional inequality**	**Overall inter-province inequality**	**Within- regional inequality**	percentage of within- regional inequality	**Between- regional inequality**	**percentage of between- regional inequality**
2019	0.0559	0.0353	63.16	0.0206	36.84	0.0556	0.0349	62.83	0.0207	37.17
2020	0.0571	0.0370	64.81	0.0201	35.19	0.0560	0.0358	63.95	0.0202	36.05
2021	0.0587	0.0412	70.09	0.0176	29.91	0.0564	0.0389	69.02	0.0175	30.98
2022	0.0575	0.0412	71.74	0.0162	28.26	0.0543	0.0381	70.27	0.0161	29.73
2023	0.0575	0.0423	76.67	0.0152	27.50	0.0552	0.0400	72.51	0.0152	27.49

**Table 5:** Regional attribution of Theil T and Theil L for health professionals in China, 2019–2023.

**Year**	**Theil T**					**Theil L**				
	**Overall inter-province inequality**	**Within- regional inequality**	**percentage of within- regional inequality**	**Between- regional inequality**	**percentage of between- regional inequality**	**Overall inter-province inequality**	**Within- regional inequality**	**percentage of within- regional inequality**	**Between- regional inequality**	**percentage of between- regional inequality**
2019	0.0457	0.0207	45.34	0.0250	54.66	0.0438	0.0196	44.7	0.0242	55.3
2020	0.0472	0.0241	50.99	0.0231	49.01	0.0454	0.0254	55.94	0.0225	49.48
2021	0.0476	0.0268	56.24	0.0208	43.76	0.0455	0.0254	55.88	0.0201	44.12
2022	0.0461	0.0272	59.04	0.0189	40.96	0.0434	0.0252	58.08	0.0182	41.92
2023	0.0447	0.0287	64.21	0.0160	35.79	0.0426	0.0270	63.36	0.0156	36.64

## 5 Discussion

### 5.1 Shortages and aging of public health workforce in China

This longitudinal study examined workforce dynamics within CDC across mainland China from 2019 to 2023. The selected timeframe offers a comprehensive view of the CDC workforce’s transformation, beginning with a pre-pandemic baseline and extending through the peak of COVID-19 response and into the recovery phase. This period is particularly valuable because it highlights how urgent public health needs can accelerate structural and policy changes, informing strategies for future workforce planning and crisis management. By examining these years, the study provides critical insights into how emergency conditions influence long-term workforce development and resource allocation. Analytical findings have revealed a notable upward trajectory in CDC personnel during the study period, contrasting with the preceding downward trend observed between 2016 and 2019 (37). Specifically, the COVID-19 crisis appears to have driven both immediate and structural changes: Mainly impact is the emergency response expansion, cause epidemic containment requirements likely prompted temporary workforce surges during public health emergencies. More importantly, it results in strategic resource reinforcement. Heightened institutional prioritization of public health infrastructure manifested through sustained increases in dedicated funding allocations and expanded workforce quotas (18, 38). The observed workforce expansion patterns align temporally with major pandemic containment phases, suggesting a direct correlation between epidemic pressures and human resources mobilization strategies.

Although the number of CDC human resource in China has shown an upward trend, there remains a gap between the current number of CDC personnel and the national target outlined in the 2015 National Healthcare Service System Planning Outline (2015–2020), which set the target at 1.75 staff per 10,000 population (8). By 2023, the gap is estimated to be 15,529 staff members, based on China’s population of 1.412 billion. Furthermore, the emerging challenge lies in achieving the updated target of 250,000 CDC staff by 2025, as outlined in recent policy directives. Current projections indicate a remaining gap of approximately 20,000 personnel (equivalent to 8% of the 2025 target) (14), necessitating an annual recruitment increase of 6.7% from 2023 levels to ensure timely compliance. Comparatively, the United States had approximately 200,000 CDC employees in 2019, with a population of 328.33 million, yielding a staffing density of 6.09 staff per 10,000 population—significantly higher than China’s (39).

Additionally, China’s CDC human resource faces an aging trend, with over 39.82% of workers aged 45 or older in 2023, and 11.06% over the age of 55. This presents a challenge as many key personnel are nearing the age of retirement, which underscores the need for a structured approach to developing a younger generation of talent. China’s current statutory retirement age is 60 for male employees, 50 for female employees, and 55 for female cadres. Despite the fact that in 2024, a document was issued that China will take 15 years to gradually delay the retirement age of male employees to 63 years old, and that of female employees to 55 and 58 years old (40), but in the future, the issue of a significant number of key personnel reaching retirement age within the same time frame will still pose a challenge.

The United States also faces significant workforce sustainability challenges stemming from accelerating demographic aging. Empirical evidence from the 2021 Public Health Workforce Interests and Needs Survey (PH WINS) reveals that approximately 20% of American public health workforce anticipate retirement within the next 5 years (20). This impending exodus of seasoned experts poses substantial institutional risks, as sector-specific analyses suggest the concentrated retirement of senior public health administrators will precipitate critical knowledge attrition and erosion of operational competencies (41). Such projections underscore the imperative for developing robust succession management frameworks within public health governance structures. Notably, this emerging workforce crisis remains understudied within China’s academic discourse, presenting a crucial research gap that demands systematic investigation. Future scholarship should prioritize comparative analyses of intergenerational knowledge transfer mechanisms and sustainable human capital strategies in public health systems.

### 5.2 Long tenure of CDC human resource in China

A striking feature of China’s public health workforce is the notably long tenure, with 46.25% of personnel having over 20 years of service as of 2023. In comparison, in the United States, only 13% of public health workers had comparable experience in 2021 (20). This difference is striking and suggests that the structure and dynamics of China’s public health workforce are distinct from those in other countries, particularly in the context of disease control and prevention.

One primary reason for the long tenure among Chinese CDC staff lies in the country’s unique staffing system, known as “bianzhi” (42). This system, which is deeply embedded in China’s civil service, establishes fixed personnel quotas for each institution, including CDCs. Bianzhi guarantees job stability by limiting fluctuations in workforce numbers, meaning that once employees are hired, they often remain in the system for long periods, with limited turnover. This stability can be highly attractive to employees, especially in a competitive job market where job security and benefits are highly valued.

Furthermore, this system discourages the constant hiring and firing seen in many Western countries, contributing to a workforce with substantial experience and institutional knowledge. The long tenure is also facilitated by the relative lack of mobility within the system. While this can promote expertise and a deep understanding of public health challenges, it also means that younger or more dynamic talent may face difficulties entering the system, potentially stifling innovation and adaptability. Additionally, the hierarchical nature of China’s public health system may reinforce long tenure. Senior positions, which are often held by long-serving personnel, are highly respected, and career progression within the CDC often relies on internal promotions rather than external recruitment. This could contribute to a workforce that, while experienced, may be less flexible in adapting to emerging public health challenges.

Thus, while the long tenure of China’s CDC workforce is a testament to the stability and experience within the system, it may also highlight structural challenges related to flexibility and the infusion of new ideas.

### 5.3 Staffing inequity persists in China

The results of the Gini coefficient showed that the Gini coefficient by geographic distribution was above the 0.4 warning line, the Gini coefficient by economic distribution was near the 0.4 warning line, and the Gini coefficient by population distribution was below 0.2. Actually, China’s healthcare system demonstrates systemic spatial inequities in health resource allocation, characterized not only by disparities in CDC workforce distribution but also by profound imbalances across hospital networks, primary care facilities, and critical infrastructure deployment (43–45), reflecting structural deficiencies in equitable resource governance. Currently, China’s health resource including public health staffing is based primarily on population distribution (46), and although the state recommends that staffing ratios be appropriately increased in areas with large geographic areas and low population densities, there are difficulties in implementing this, as many provinces in China, such as Tibet, Inner Mongolia, and Qinghai, where low population densities coexist with economic backwardness, and where the local government does not have a high level of finances, are not sufficiently attractive to public health personnel, which increases the workload of public health personnel, especially those at the grassroots level, and it is recommended that emphasis be placed on strengthening the construction of public health personnel in areas where sparsely populated and economically backward areas coexist.

The results of the Theil index showed that within-region inequality is the primary driver of staffing disparities. Some earlier studies have also shown that within-provincial differences are greater than inter-provincial differences, and that the equity of economic development and CDCs human resource allocation between different counties cannot be ignored (47). It is important to focus on coordinated regional development, as well as coordinated development among the provinces within the region.

Some studies have shown that the proportion of public health financial expenditures going to CDC institutions has shown a declining trend, from 45.87% in 2010 to 18.28% in 2022. At the same time, CDC construction was largely funded by local government finances, with little construction responsibility borne by the central government over the past 10 years, which is closely related to the inequity of public health staffing (38).

## 6 Conclusion

Despite an increase in China’s public health workforce from 2019 to 2023, driven by post-pandemic capacity building efforts, systemic challenges persist in achieving sustainable development goals:

First, quantitative gaps remain substantial, with a 15,529 personnel deficit against the 2015 national standard (1.75/10,000 population) and an 8% shortfall (equivalent to 20,000 personnel) for the 2025 target of 250,000 CDC workforce. Compounding this, aging demographics threaten operational continuity—39.82% of workers aged ≥45 years and 11.06% nearing retirement (≥55 years) in 2023.

Second, structural inequities undermine system resilience. Geographic disparities (Gini coefficient > 0.4) and intra-regional imbalances (Theil Index contributions: 64–77% within Eastern/Central/Western zones) reveal fragmented resource allocation.

Third, the misalignment between policy and practice exacerbates challenges. Despite mandating workforce expansion, current incentive structures fail to address root causes: CDC salaries lag behind tertiary hospitals. Meanwhile, the imperfect salary incentive mechanism has fallen into the dilemma of excessive egalitarianism, resulting in the coexistence of insufficient attractiveness of disease control institutions to personnel and high attribution rates. To achieve sustainable development, a three-pillar strategy is imperative: (1) Workforce revitalization: Optimize salary and incentive mechanisms, coupled with accelerated recruitment of young professionals annually through national public health scholarship programs (2) Equity-driven redistribution: Establish a dynamic resource allocation matrix using AI-powered demand forecasting, prioritizing understaffed regions (3) Capacity modernization: Launch cross-regional competency centers to standardize training, leveraging digital platforms to reduce skill variance.

Future reforms must synchronize quantitative expansion with qualitative upgrades, recognizing that workforce sustainability hinges equally on numerical targets and systemic equity.

## 7 Strengths and limitations

This study offers several significant strengths, most importantly its novelty, to our knowledge, it addresses a research gap by providing a comprehensive description of China’s current situation of CDC human resource, estimating the temporal trends and recognizing the equity by geographic, economic and population distribution (48). Moreover, this study used data from the CNBIS, a nationally annual organization-based administrative survey. Therefore, the findings of this study exhibit strong national situation of public health workforce, underpinned by the reliability and high-quality of CNBIS data. Besides, the five-year analytical timeframe (2019–2023), proves strategically significant as it bridges the pre-pandemic baseline (2019) and post-pandemic recovery phase (2020–2023). Such temporal positioning enables systematic evaluation of COVID-19’s structural impacts on workforce deployment mechanisms within China’s Centers for Disease Control and Prevention (CDC). The longitudinal design effectively captures institutional adaptations during public health emergencies, offering empirical evidence for optimizing human capital strategies in crisis preparedness frameworks.

However, the study also has limitations. First, the analysis is confined to quantitative data on workforce numbers due to the nature of data disclosure. This limitation precludes an evaluation of qualitative aspects, such as personnel competence or performance, which may also influence workforce dynamics. Second, while the five-year timeframe is strategically significant, it may not represent typical operational conditions, as the crisis-driven environment likely influenced recruitment patterns and resource distribution in ways that differ from non-emergency periods. Future research could integrate qualitative measures and extended timeframes to provide a more comprehensive understanding of both workforce quality and distributional equity.

## Data Availability

The datasets presented in this article are not readily available because the data utilized in this study were sourced from the China CDC and are not publicly available. Access to these data is subject to approval and can be requested directly from the China CDC by interested researchers. Requests to access the datasets should be directed to website@chinacdc.cn.
